# Enhancing genetic modification in recalcitrant plants: An investigation in chili (*Capsicum annuum*) through the optimized tape sandwich protoplast isolation and polyethylene glycol-mediated transfection

**DOI:** 10.5511/plantbiotechnology.24.0613a

**Published:** 2024-12-25

**Authors:** Hanggara Aji Sakti Mahambara Padma Negara, Rizkita Rachmi Esyanti, Iriawati Iriawati, Santiago Signorelli, Rinda Kirana, Karlia Meitha

**Affiliations:** 1 School of Life Sciences and Technology, Institut Teknologi Bandung, Bandung 40132, Indonesia; 2 School of Agriculture, Universidad de la República, Montevideo 11200, Uruguay; 3 Horticulture Research Center, National Research and Innovation Agency, Bogor 16915, Indonesia

**Keywords:** chili, CRISPR/Cas9, polyethylene glycol, protoplast isolation, transfection

## Abstract

Chili presents challenges for *Agrobacterium*-mediated transfection due to its highly recalcitrant nature. One way to overcome this challenge is by using PEG-mediated transfection of protoplasts, which enhances the likelihood of successfully introducing transgenes into the cells. The tape sandwich method for isolating chili leaf protoplasts was optimized by adjusting enzyme concentrations and incubation duration, resulting in a high yield of 1.3×10^6^ cells ml^−1^ per 0.1 g of leaves. The efficiency of transfecting GFP-encoding plasmids and Cas9 protein using PEG molecules of different sizes was also examined. The highest plasmid transfection efficiency was achieved with 5 µg of plasmid in 50 µl^−1^, with an average efficiency of 48.71%. For Cas9 protein transfection, the most effective treatment involved using 1000 µg of protein in 100 µl^−1^, mediated by 40% PEG 4000, resulting in an average efficiency of 2.94% due to protein aggregation. Nevertheless, this optimized protocol reduces the time required for chili protoplast isolation and enhances plasmid transfection efficiency by nearly 50%.

Advancements in biotechnology improves the quality of important crops by modifying their genetic makeup. One common technique involves using *Agrobacterium* to introduce specific genes into plants. However, many plants exhibit resistance to this process, known as recalcitrance (Tiwari et al. 2022). Chili plants (*Capsicum annuum* L.) in particular, are highly recalcitrant, making it challenging to genetically modify them with low success rates across different varieties (Heidmann et al. 2011; Kumar et al. 2012). Therefore, it is necessary to optimize the transfection method for specific chili cultivars through empirical experimentation. Transfection of chili protoplasts, which removes barriers like cell walls, is considered as an alternative in facilitating the introduction of external DNA, RNA, or proteins into cells, enhancing gene transfer (Reed and Bargmann 2021).

PEG-mediated transfection of protoplasts is a safer and more practical technique for introducing exogenous polynucleotides and proteins into plant cells compared to other methods like biolistics. It causes minimal damage to cells and is an affordable and efficient strategy that doesn’t require specialized organisms or equipment (Jeon et al. 2007; Lacroix and Citovsky 2020). The application of PEG enhances the efficacy of transfection by facilitating the permeabilization of cell membranes in protoplasts (Guo et al. 2022; Pan et al. 2022). Regenerating plants from engineered protoplasts helps avoid chimerism commonly observed in transgenic calli (Reed and Bargmann 2021). In this study, we optimized the tape sandwich method to prepare protoplasts from chili leaves and evaluated the transformation efficiency following transfection with plasmid (expressing GFP) and Cas9 protein (fused with GFP).

Seeds of chili ‘Inata Agrihorti’ were obtained from the Indonesia Vegetable Research Institute (IVegRI) in Lembang, West Java. These mature seeds were sown onto soil and grown in a room with a 16 h light and 8 h dark photoperiod. The leaves used as protoplast sources were approximately 5.5–6 cm long and 2–2.5 cm wide, with a fresh weight of ±0.1 g (FW) per sample leaf. We used the pK7WGF2 plasmid (Karimi et al. 2002) containing the GFP:NLS gene under a 35S promoter to assess the efficiency of plasmid transfection. Meanwhile, the Cas9:NLS:GFP protein was recombinantly produced from 4xNLS-pMJ915v2-sfGFP plasmid obtained from the Addgene Plasmid, Repository #88921 (Staahl et al. 2017).

Chili leaf protoplasts were isolated using the modified tape-*Arabidopsis* sandwich method (Wu et al. 2009). Healthy chili leaves were sprayed with 70% ethanol, cleaned using a clean tissue and then weighed. The upper and lower epidermis of the leaves were covered using masking tape to create a ‘sandwich’. Subsequently, the lower epidermis was carefully peeled off as much as possible, leaving only the mesophyll and upper epidermis. The leaves were then submerged in an enzyme solution (20 mM MES pH 5.7, 400 mM mannitol, 20 mM KCl, 1–2% (0.1–0.2 g for 10 ml) cellulase Onozuka R-10 and 0.2–0.4% (0.02–0.04 g for 10 ml) macerozyme R-10 (PhytoTech Labs, USA), 10 mM CaCl_2_, and 0.1% (w/v) BSA) that had been previously filtered using a 0.45 µm sterile microfilter. The leaf bath was then agitated at 50 rpm for 1 to 3 h. After incubation, the leaves were gently shaken for 5–10 min to maximize the protoplast release, and the cell suspension was filtered using 80 µm nylon sieves while being rinsed with W5 solution (5 mM glucose, 154 mM NaCl, 125 mM CaCl_2_, 5 mM KCl, and 2 mM MES pH 5.7). The protoplasts were harvested by centrifugation at 50×g for 2 min, and the supernatant was removed as much as possible; this was performed twice. The protoplast pellet was resuspended in 1 ml of W5 solution and kept on ice for 15 min while the yield was counted using a hemocytometer.

The plasmid and Cas9 protein were transformed into previously isolated protoplasts using PEG-mediated method (Brandt et al. 2020; Yoo et al. 2007). The supernatant was removed from the settled protoplast suspension, and the protoplasts were suspended in MMG solution (4 mM MES pH 5.7, 400 mM mannitol, and 15 mM MgCl_2_) to a concentration of 5×10^4^ cells in 100 µl^−1^. Next, 50 µl of plasmid (2.5–5 µg) or 100 µl Cas9:NLS:GFP protein (10, 100, and 1000 µg) was added to a 2 ml round-bottomed microcentrifuge tube, followed by the addition of 100 µl of protoplasts (5×10^4^ cells) and 150 µl of 40% PEG 4000 or PEG 6000 solution (100 mM CaCl_2_, 200 mM mannitol, and 40% (w/v) PEG 4000 or 6000). The mixture was gently tapped until thoroughly combined. The mixture was incubated at room temperature for 30 min, after which 600 µl of W5 solution was added to stop the transfection process. The protoplasts were harvested again by centrifugation at 100×g for 1 min, and the supernatant was discarded. The protoplast pellet was then resuspended in 250 µl of W5 solution and incubated overnight in 24-well plates coated with 5% (v/v) BSA in the dark.

GFP fluorescence in protoplasts was observed using the Olympus Laser Scanning Microscope FV1200, and the images were processed using the Olympus Fluoview ver. 4.2a. The filters utilized were EGFP for detecting GFP fluorescence and Alexa Fluor 647 for detecting chloroplast autofluorescence. DAPI staining was performed based on the method by (Pasternak et al. 2015) to confirm the successful entry of Cas9 protein into the protoplast nucleus, and its visualization was performed using a DAPI filter on a confocal microscope. The percent transfection efficiency (%) was calculated by dividing the number of fluorescent protoplasts by the total number of observed protoplasts multiplied by 100%. All statistical analysis was performed using Prism 9 software (GraphPad, US). The one-way ANOVA test with Tukey’s *posthoc* test (parametric) or the Kruskal–Wallis test with Dunn’s *posthoc* test (non-parametric) was used to determine statistical significance. The *p* values were reported in GraphPad style, where (ns) indicates *p*>0.05, (*) indicates *p*≤0.05, (**) indicates *p*≤0.01, (***) indicates *p*≤0.001, and (****) indicates *p*≤0.0001.

In a preliminary test, two modified methods for isolating chili leaf protoplasts were compared: a simplified cutting method without vacuum infiltration and the tape sandwich method using masking tape (both performed with 2% cellulase and 0.4% macerozyme). The goal was to determine the method that resulted in protoplast isolates with minimal cell damage, ease of execution, and the shortest incubation period to yield more than 10^6^ cells ml^−1^ (data not presented). Visually, the cutting method yielded protoplasts with a higher amount of cell debris, despite undergoing filtration and being washed twice with W5 solution. In contrast, the tape sandwich method had fewer damaged cells (Figure 1A, B). However, due to the vascular structures of the chili leaf, the masking tape could not adhere to the epidermal tissue surrounding the leaf veins, resulting in incomplete tissue removal in that area (Figure 1C, D). This method may be more efficient when performed on young leaves or cotyledons, as they lack thickened vascular structures.

**Figure 1:**
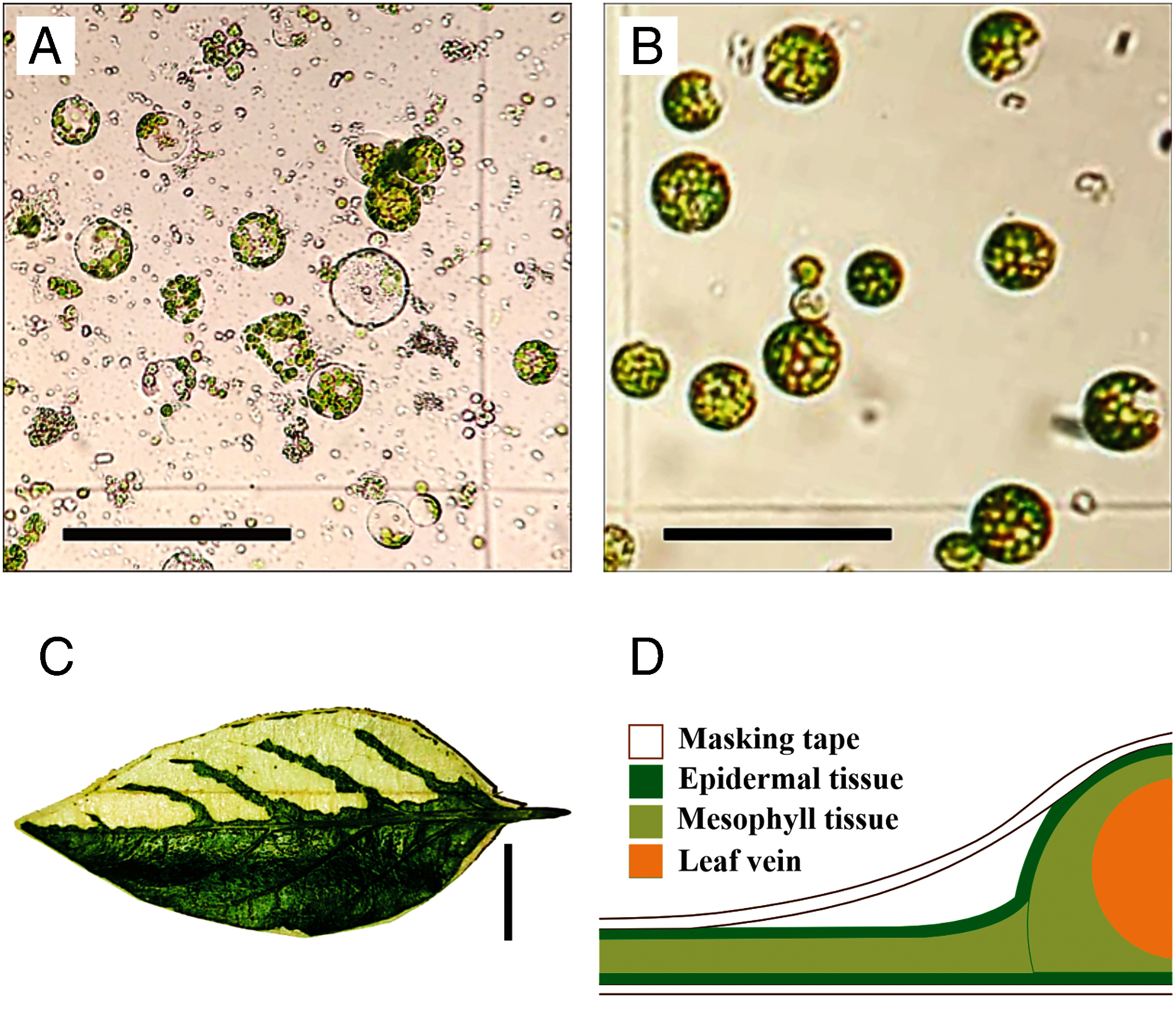
Figure 1. Protoplast isolation preliminary test of chili cv. ‘Inata Agrihorti’. Comparison of protoplasts obtained through cutting (A) and tape sandwich (B) methods. The cutting method produced more cell debris than the tape sandwich (scale bar: 100 µm). (C) Comparison between leaf sections where the lower epidermis was removed using masking tape (white) and sections where the lower epidermis was still intact (green) after being submerged in enzyme solution (scale bar: 1 cm). This picture also shows the irremovable epidermal tissue along the leaf veinal structures. (D) The diagram shows how the masking tape cannot adhere to the epidermal tissue. The diagram is not drawn to scale as it is a simplified version of the tape sandwich-making process.

Based on the test results, chili leaf protoplasts were isolated using the tape sandwich method. Figure 2A shows that the protoplast yield was influenced by the concentration and combination of enzymes, as well as the duration of the incubation period. When leaves were immersed in varying enzyme concentrations, the average protoplast yield ranged from 1.4×10^5^–1.3×10^6^ cells ml^−1^ per 0.1 g of sample leaf with the optimal yield was achieved by immersing the leaves in 2% cellulase and 0.4% macerozyme solution for 3 h (Figure 2B). Macerozyme is crucial in breaking down the middle lamella layer, facilitating cell separation (Agrios 2005; van den Brink and de Vries 2011). Increasing the macerozyme concentration enhances the efficiency of cellulase in breaking down the cell walls of mesophyll cells. It is recommended to use higher concentrations of both cellulase and macerozyme for optimal results, as they synergistically degrade polysaccharides (Berlemont 2017).

**Figure 2:**
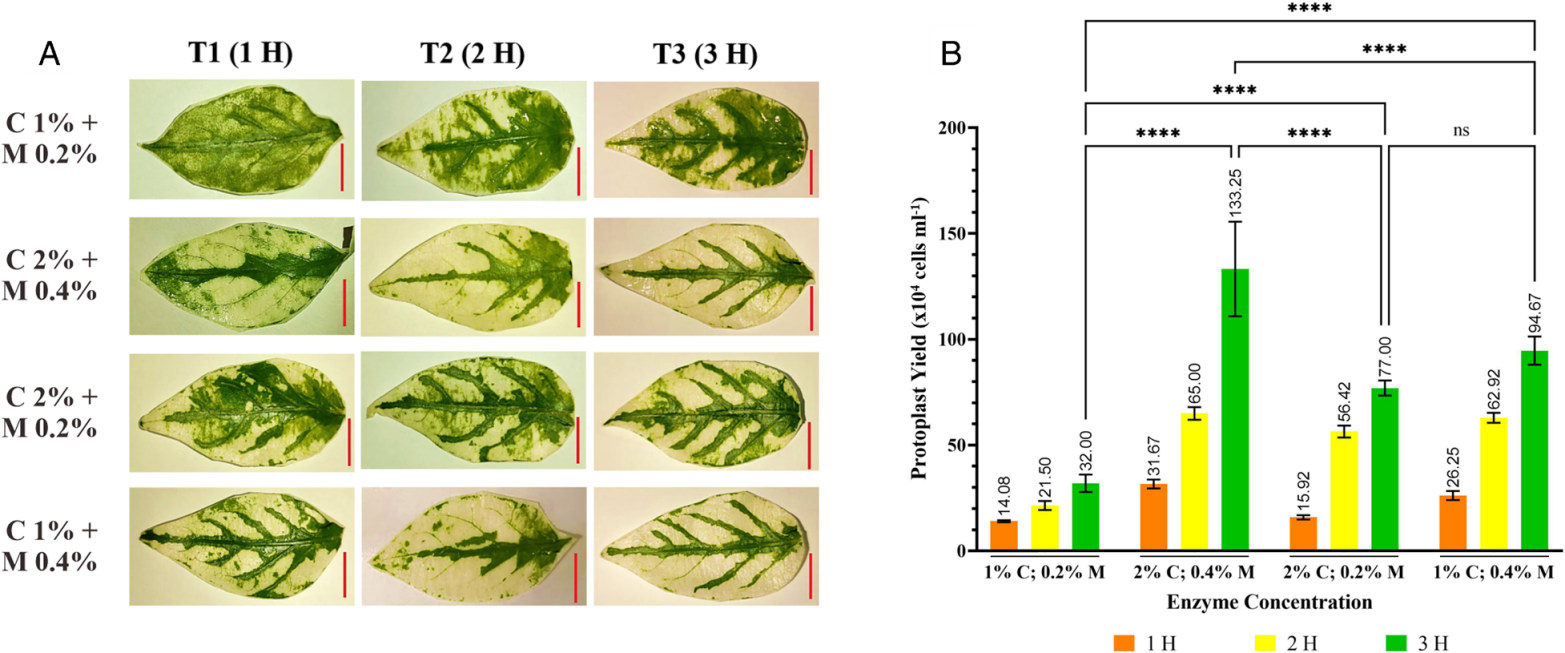
Figure 2. Enzyme concentration and incubation duration affect the isolation efficiency of the chili protoplasts. (A) Comparison of the chili leaves appearance after being submerged in different enzyme solution and durations (scale bar: 1 cm). T denotes the incubation duration. (B) The bar chart shows the yield of isolated protoplasts for every 0.1 g of chili leaves under different treatments. The cellulase and macerozyme concentrations used are represented by % C and % M. (ns) denotes non-significant differences, while (****) indicates significant differences where *p*<0.0001.

This experiment resulted a high protoplast yield in a relatively short time. When converted to grams of fresh weight (gFW) of leaves, the use of a combination of 2% cellulase and 0.4% macerozyme within three hours of incubation is estimated to result in the production of 1.3×10^7^ protoplasts. Previous studies on chili protoplast isolation required over 12 h of incubation to obtain yields higher than 10^8^ cells (Jeon et al. 2007; Prakash et al. 1997). Although the protoplast yield in this study was lower compared to those studies, it was considered sufficient for the transfection experiment, which typically requires 10^4^–10^6^ cells per reaction (Brandt et al. 2020; Jeon et al. 2007; Kim et al. 2020; Wu et al. 2009; Yoo et al. 2007). Moreover, this experiment offers a shorter incubation period and requires minimal equipment compared to the cutting method, saving both research time and cost.

Successful transfection with the pK7WGF2 plasmid was confirmed by nucleus-localized green fluorescence (Supplementary Figure S1A, B). The data on transfection efficiency showed that the difference in PEG molecular weight did not significantly impact the percentage of transfection efficiency. But, it is known that prolonged incubation of protoplasts in PEG 6000 can cause various stresses that may disrupt cell membrane stability, making the protoplasts are more susceptible to lysis (Beranek et al. 2007; Filek et al. 2012; Hahn-Hagerdal et al. 1986; Mohammadkhani and Heidari 2007; Wang et al. 2021; Wu et al. 2020). Additionally, PEG 6000 is considered a more potent inducer of protoplast fusion compared to PEG 4000 (Supplementary Figure S2). The presence of cations such as Ca^2+^ in the PEG buffer is also known to trigger protoplast aggregation (Ahmed et al. 2021; Davey 2017). These factors are believed to affect the accuracy of protoplast yield counting.

The transfection efficiency was more affected by the varying concentrations of plasmids used, with the use of 5 µg 50 µl^−1^ of plasmid and 40% PEG 4000 resulting in the highest efficiency average of 48.71% (Figure 3A). These findings indicate that an increase in plasmid concentration corresponds to a higher transfection efficiency, confirming the association between the quantity of transfected plasmid concentration and the level of gene expression (Jeon et al. 2007). The obtained results in this study are considered promising, as they exceed most of the biolistic- and *Agrobacterium*-mediated transfection frequencies reported in chili (Aarrouf et al. 2012; Chee et al. 2018; Hasnat et al. 2008; Li et al. 2003; Mahto et al. 2018; Zhang et al. 2018). The GFP fluorescence, with an average efficiency of almost 50%, also implies that our optimized protoplast transfection method is suitable for facilitating transfection in chili ‘Inata Agrihorti’.

**Figure 3:**
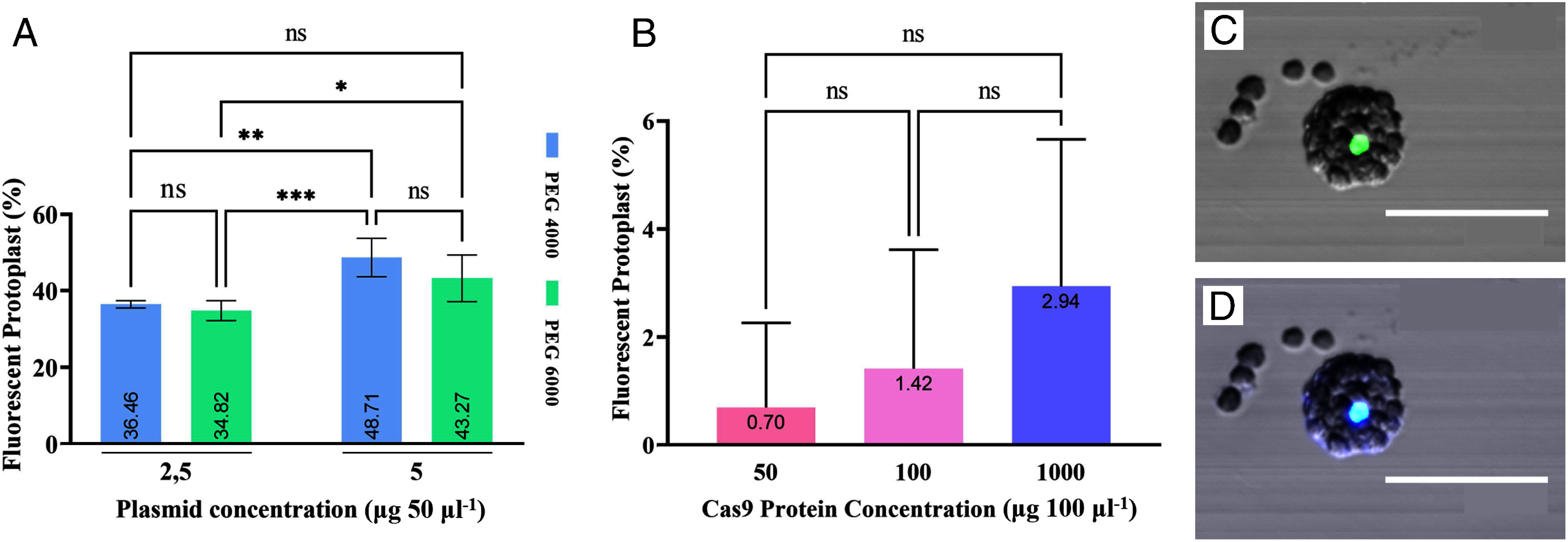
Figure 3. Protoplast transfection efficiency in chili cv. Inata Agrihorti protoplast. (A) The efficiency of the plasmid transfection was not affected by the molecular weight of PEG, but rather by the concentration of plasmid used. (ns) not significantly different; (*) significantly different at *p*<0.05; (**) *p*<0.01; (***) *p*<0.001. (B) All Cas9 protein concentration treatments showed no significant difference in results. (ns) not significantly different. Double staining imaging of fixed protoplasts using confocal microscopy: (C) GFP filter and (D) GFP+DAPI. Overlapping staining indicates successful protein localization within the nucleus (scale bar: 50 µm).

We also evaluated the effectivity of Cas9:NLS:GFP delivery into the nucleus by measuring transfection efficiency through GFP fluorescence (Figure 3B, C) and confirming it with DAPI double staining (Figure 3D). Within 18–20 h after transfection, Cas9 protein was detected and localized into the nucleus. Interestingly, different concentrations of Cas9 protein had minimal impact on transfection efficiency, except for an increase in the detection of fluorescent bodies outside the protoplast (Supplementary Figure S3A, B). The efficiency was relatively low compared to the concentration of Cas9 protein used. Transfection efficiencies of only 2.94% and 0.70% were achieved with protein treatments of 1000 µg 100 µl^−1^ and 50 µg 100 µl^−1^, respectively. This is in contrast to a previous study conducted by Subburaj et al. (2022), which achieved a transfection efficiency of 38% using only 10 µg µl^−1^ of Cas9 protein within 18 h in soybean. These findings suggest that other factors may be influencing the success of transfection in this experiment.

The low efficiency could be attributed to the aggregation of the Cas9:NLS:GFP protein. Under confocal microscopy, fluorescent bodies larger than the typical size of protoplasts from chili ‘Inata Agrihorti’ were observed, indicating the presence of protein aggregates (Supplementary Figure S4A, B). The aggregation of Cas9:NLS:GFP size was up to 50 µm, making it difficult to be transfected into protoplasts. When an excessive protein suspension is added, the likelihood of aggregate formation also increases. Consequently, the detection of protein aggregates is higher in the treatment with 1000 µg 100 µl^−1^ protein compared to other treatments. Furthermore, during the washing process, protein aggregation can accumulate in mass and precipitate alongside the protoplasts, leading to their detection during confocal observation even after washing. To mitigate protein aggregation and improve transfection efficiency, it is necessary to further optimize the production of Cas9:NLS:GFP and its transfection conditions.

In summary, this study presents an efficient approach to overcome the challenge of genetically modifying chili plants through PEG-mediated protoplast transfection. The tape sandwich method was optimized to effectively isolate protoplasts by removing the lower leaf epidermis. By combining high concentrations of cellulase and macerozyme and incubating for three hours, a high yield of protoplasts suitable for transfection studies was achieved. However, optimization process of protoplast isolation might be required if using different chili genotype. The efficiency of plasmid transfection was primarily influenced by the concentration of the plasmid rather than the molecular weight of PEG. However, in the transfection by protein, protein aggregates hindered the successful transfection of Cas9:NLS:GFP protein. To overcome this obstacle, it is recommended to optimize protein production and transfection conditions. Also, a higher stringency in the sterilization preparation is highly recommended if the experiment involves stable transformation.

## Abbreviations

Cas9: CRISPR-associated protein 9
DAPI: 4′,6-diamidino-2-phenylindole
GFP: green fluorescent protein
NLS: nuclear localization signal
PEG: polyethylene glycol

## Description of Supplementary Files

Supplementary Figure S1. GFP expression in chili cv. Inata Agrihorti protoplast.

Supplementary Figure S2. Protoplasts transfected by PEG 4000 or 6000.

Supplementary Figure S3. Detection of Cas9 protein aggregates outside the protoplasts.

Supplementary Figure S4. Large aggregates of Cas9: NLS: GFP fusion protein.
