# Supplementary material for: Enhancing genetic modification in recalcitrant plants: An investigation in chili (Capsicum annuum) through the optimized tape sandwich protoplast isolation and polyethylene glycol-mediated transfection
Source: Plant Biotechnol (Tokyo). 2024 Dec 25;41(4):459–64. doi: 10.5511/plantbiotechnology.24.0613a (PMC11897716; doi:10.5511/plantbiotechnology.24.0613a)
Supplement: Supplementary Data [file plantbiotechnology-41-4-24.0613a-s001.pdf]

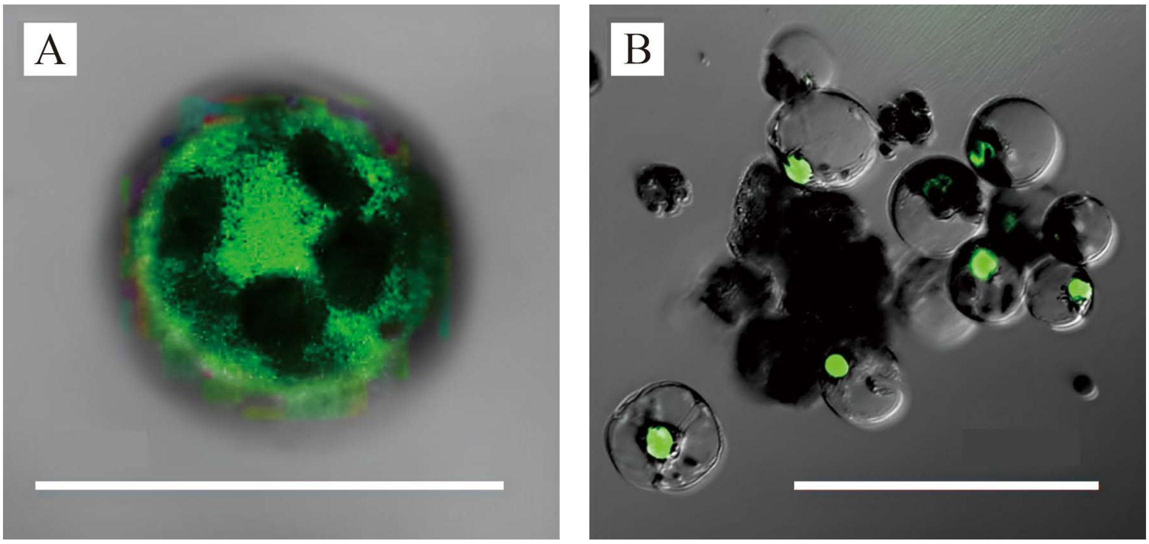

Supplementary Figure S1. GFP expression in chili cv. Inata Agrihorti protoplast. A-B Difference in imaging of protoplasts expressing (A) GFP (scale bar: 50 μm) and (B) GFP:NLS plasmids (scale bar: 100 μm). The large, dark-colored spheres inside the protoplasts are chloroplasts.

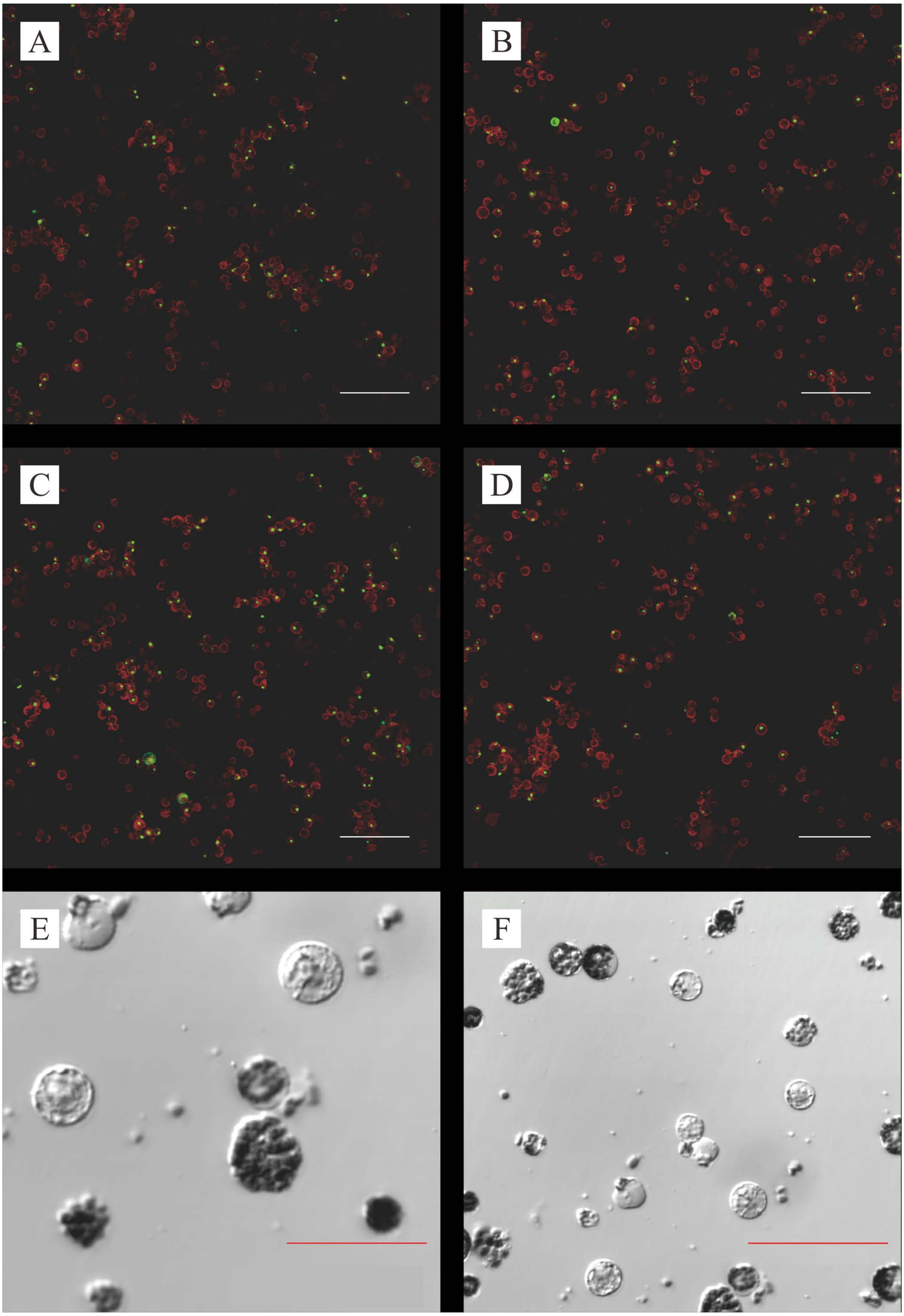

Supplementary Figure S2. Protoplasts transfected by PEG 4000 or 6000: (A) 2.5 μg 50 μl^{-1} plasmid in PEG 4000; (B) 2.5 μg 50 μl^{-1} in PEG 6000; (C) 5 μg 50 μl^{-1} in PEG 4000; (D) 5 μg 50 μl^{-1} in PEG 6000 (scale bar: 200 μm). (E) Non-transfected protoplast in PEG 4000 (scale bar: 80 μm); (F) Non-transfected protoplast in PEG 6000 (scale bar: 100 μm).

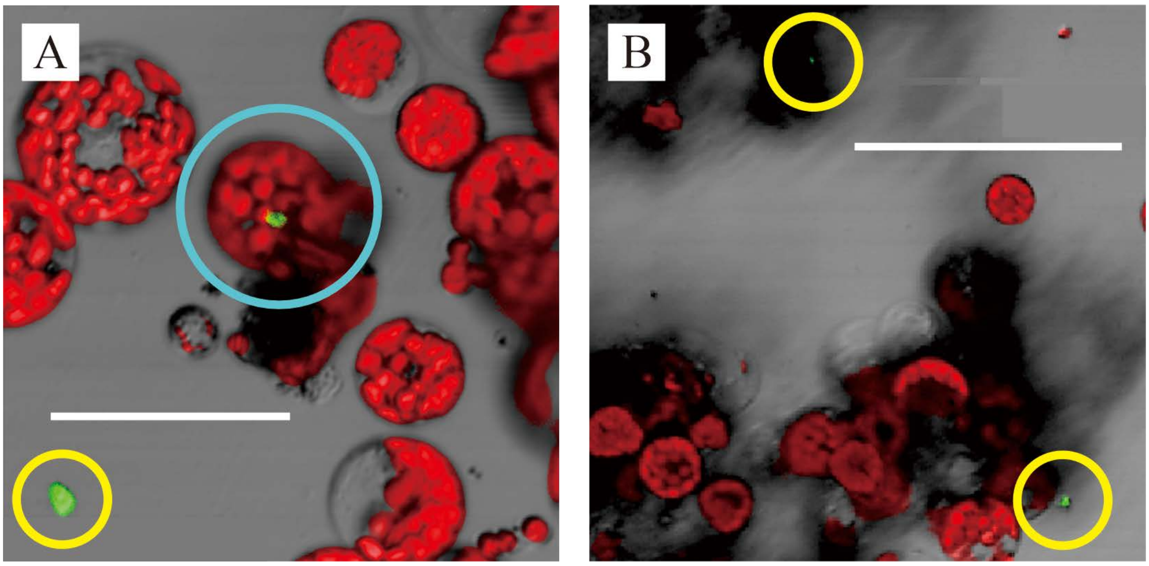

Supplementary Figure S3. Detection of Cas9 protein aggregates outside the protoplasts. (A) The Cas9:NLS:GFP aggregates from 1000 μg 100 μl^{-1} concentration treatment are found inside protoplasts (scale bar: 50 μm). (B) Some protein aggregates are found outside the protoplast (scale bar: 100 μm). Green = GFP fluorescence; red = chloroplast autofluorescence; blue circle = transfected protoplasts; yellow circle = Cas9:NLS:GFP aggregates.

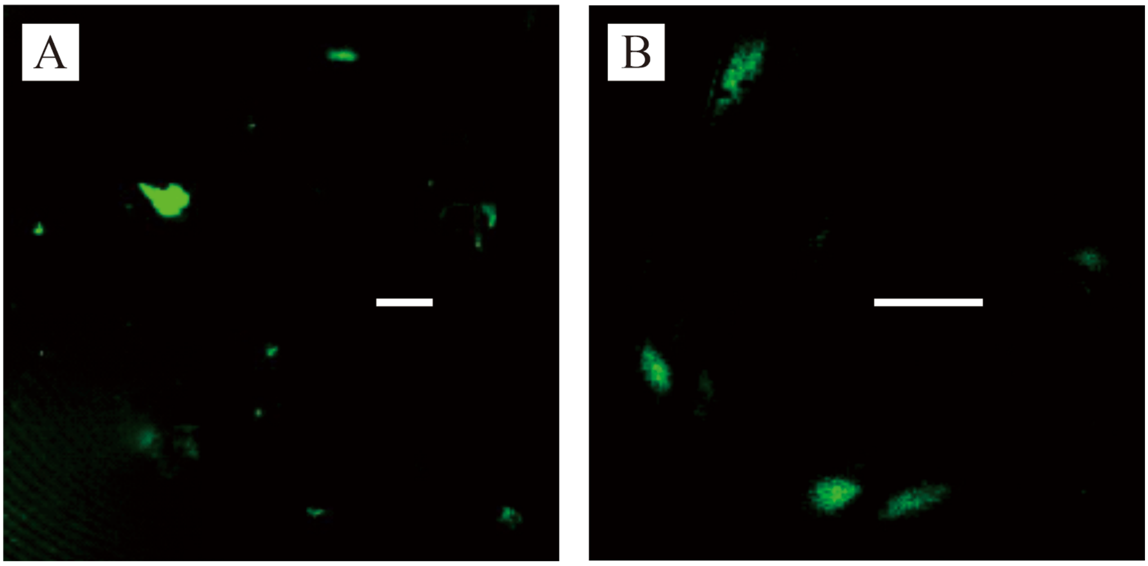

Supplementary Figure S4. Large aggregates of Cas9:NLS:GFP fusion protein. (A—B) Fluorescence imaging of the Cas9:NLS:GFP suspension revealed the presence of large protein aggregates, indicating that the protein increased size hindered the transfection process (scale bar: 50 & 100 μm).
